# Context Dependent Effect of Landscape on the Occurrence of an Apex Predator across Different Climate Regions

**DOI:** 10.1371/journal.pone.0153722

**Published:** 2016-04-28

**Authors:** Go Fujita, Atsuki Azuma, Jun Nonaka, Yoshiaki Sakai, Hatsumi Sakai, Fumitaka Iseki, Hiroo Itaya, Keita Fukasawa, Tadashi Miyashita

**Affiliations:** 1 Laboratory of Biodiversity Science, School of Agriculture and Life Sciences, University of Tokyo, Yayoi 1-1-1, Bunkyo-ku, Tokyo, 113–8656, Japan; 2 Faculty of Agriculture Environmental Sciences for Sustainability, Iwate University, Ueda 3-18-8, Morioka city, Iwate, 020–8550, Japan; 3 Goshawk Protection Fund, Hanawada, 2-5-1, Utsunomiya city, Tochigi, 320–0027, Japan; 4 Association for gray-faced buzzards in Inzai, Kigari 3-9-1, Inzai city, Chiba, 270–1359, Japan; 5 Institute for endangered species, Sadanomi, 750–2, Oita city, 879–0122, Japan; 6 Ryokusei Kenkyujo, Kojimacho, 2-40-10, Chofu city, Tokyo, 182–0026, Japan; 7 National Institute for Environmental Studies, Japan, Onogawa, 16–2, Tsukuba city, Ibaraki, 305–0053, Japan; University of Sydney, AUSTRALIA

## Abstract

In studies of habitat suitability at landscape scales, transferability of species-landscape associations among sites are likely to be critical because it is often impractical to collect datasets across various regions. However, limiting factors, such as prey availability, are not likely to be constant across scales because of the differences in species pools. This is particularly true for top predators that are often the target for conservation concern. Here we focus on gray-faced buzzards, apex predators of farmland-dominated landscapes in East Asia. We investigated context dependency of “buzzard-landscape relationship”, using nest location datasets from five sites, each differing in landscape composition. Based on the similarities of prey items and landscape compositions across the sites, we determined several alternative ways of grouping the sites, and then examined whether buzzard-landscape relationship change among groups, which was conducted separately for each way of grouping. As a result, the model of study-sites grouping based on similarities in prey items showed the smallest ΔAICc. Because the terms of interaction between group IDs and areas of broad-leaved forests and grasslands were selected, buzzard-landscape relationship showed a context dependency, i.e., these two landscape elements strengthen the relationship in southern region. The difference in prey fauna, which is associated with the difference in climate, might generate regional differences in the buzzard-landscape associations.

## Introduction

Ecologists have long sought to distinguish ecological relationships that are general from those that are idiosyncratic [1]. When identified ecological relationships are applicable and constitute a general rule in a broad range, e.g. across different climate regions, these patterns can be said to have generality or transferability [2]. Lack of generality is often a serious concern for applied studies in biodiversity conservation, such as habitat evaluation procedures using habitat models [3].

Wenger & Olden [1] argued generalities of habitat models are lessen because 1) random noises may make weak relationships between response and predictor variables, which may be incorrectly interpreted as legitimate relationships, and 2) statistical associations are real in a given dataset, but do not occur across a wide range of conditions, i.e., identified relationships being context dependent. The first reason has been well studied and certain solutions were proposed—for instance, carrying out model selection procedures [4], or avoidance of modeling methods (GAM, Neural networks and Random forest) likely to generate overfitting of a relationship in a given dataset [1].

On the other hand, the second reason is poorly understood [5]. When ranges in landscape variables or landscape compositions (i.e., x-axis) differ across regions, and if there is a non-linear relationship between the occurrence of target species and these variables, we detect seemingly different relationships among regions, even though there is actually a general relationship that can be applicable across the whole regions [6]. In addition, species-landscape relationships could vary from region to region due to, for instance, climate conditions [7]. Across different climate regions, prey fauna and/or flora are likely to be dissimilar, and even if the prey species occurs in all of these regions, abundances and phenology of prey tend to be different [8]. These differences may result in context dependency of species-landscape relationships across regions. However, we have limited knowledge on how differences in landscape compositions and prey species pools affect regional differences in species-landscape relationships.

Habitat models for apex predators are suspected to have such a context dependency, caused by differences in prey availability across regions. In addition, these predators are major targets for the establishment of habitat models for the purpose of their conservation because many populations of the predators are facing with risks of extinction, and conservation activities across multiple regions are underway [9, 10]. Because apex predators require relatively large home ranges in general, and are often vulnerable to human disturbances like habitat fragmentation [11–13], we often need habitat models that encompass different regions [12]. Despite such circumstances, little is known about context dependency of habitat models for these organisms (but see [10]).

Here we focus on gray-faced buzzards *Butastar indicus*, an apex predator of farmland ecosystems in East Asia [14]. The buzzards primarily breed in a traditional, paddy-field dominated landscape in Japan. Populations of these buzzards are suspected be in decline because of human activities such as agricultural intensifications [14, 15]. In a study on the buzzards at the center of their geographical range, Momose et al. [16] showed that breeding buzzards mainly preferred areas rich in forestedge habitat. This has been suspected because of their habitat requirements, i.e., they mainly forage in two types of neighboring habitats: forests and open fields (such as paddy fields) [14]. They mainly hunt amphibians and reptiles, in paddy fields, and large insects, such as Lepidoptera larvae, in deciduous broad-leaved forests adjacent to the paddy fields [17, 18].

As explained above, we considered here that regional differences in species-landscape relationships are due to the differences in landscape characteristics [3], or due to the differences in prey availability at each landscape element across regions. There has been both published and unpublished information on prey items of breeding gray-faced buzzards [16, 19–23](Atsuki Azuma, Fumitaka Iseki, Hiroo Itaya unpublished data, see detailed descriptions in the Hypothesis section of the Methods). Data showed that prey items used by the buzzards varied across regions, although sample sizes were limited. Because geographic distributions of major prey organisms (e.g. amphibians, reptiles, and Lepidoptera) are not homogenous throughout the breeding range of the buzzards [24], it is plausible that prey fauna is variable in space.

In the present study, we investigated context dependent relationships between the occurrence of gray-faced buzzards and landscape structures. To analyze the buzzard-landscape relationships, we used datasets on nest locations from five sites, located in different regions, covering northern, central, and southern areas of their breeding range in Japan. Based on the similarities in prey items and landscape compositions across these sites, we determined several alternative ways of grouping the five sites, and examined which grouping was most effective at explaining buzzard occurrence, and how the buzzard-landscape associations changed across the groups.

## Materials and Methods

### 1. Study Sites

**Fig 1:**
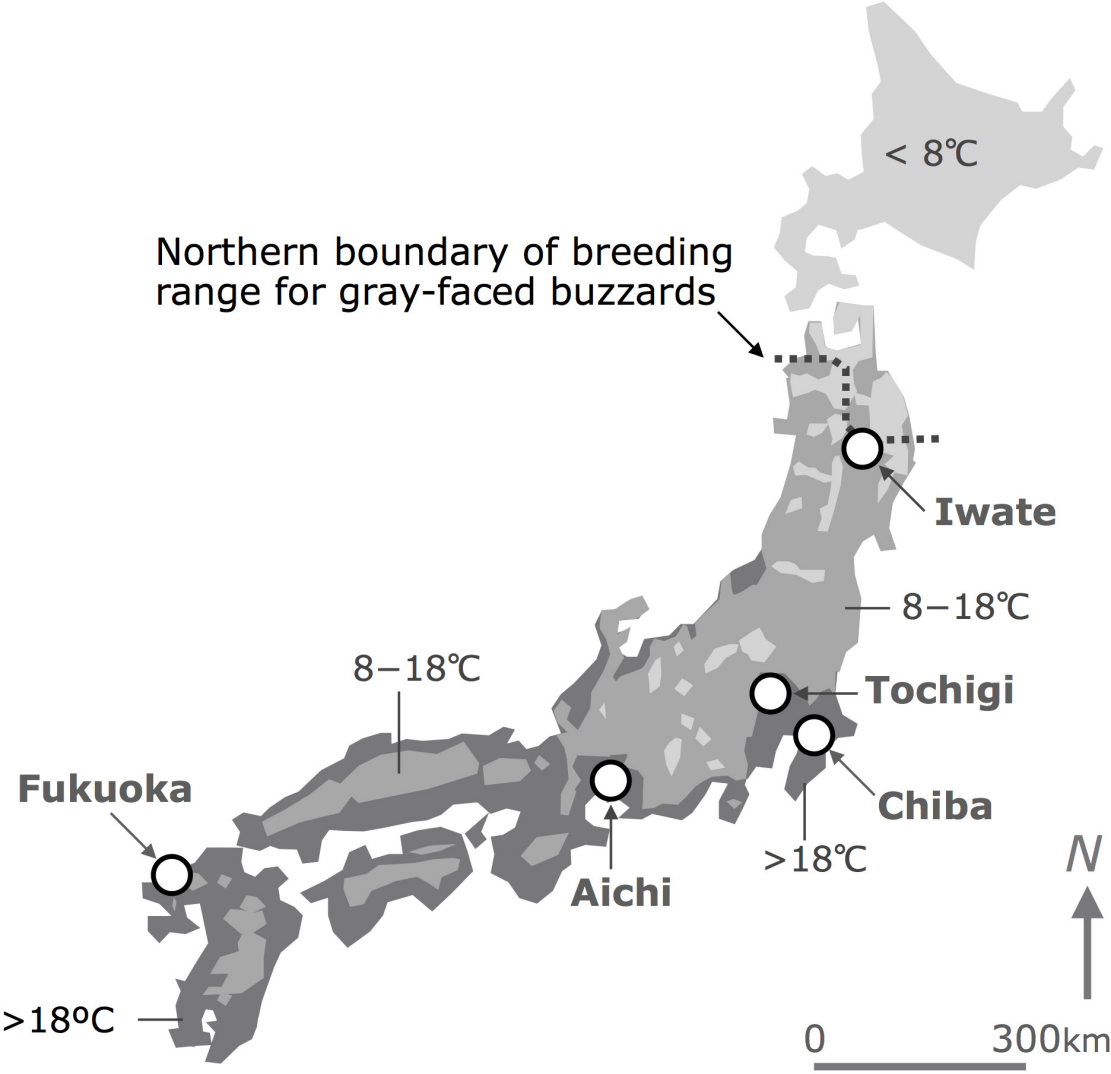
Locations of five study-sites. Average temperatures (°C) in May of 2010 are shown. We draw the map based on a map provided by Japanese Meteorological Agency [25].

Investigations for locating breeding nests of gray-faced buzzards were carried out at five sites (Fig 1). Three sites were located within the central part of the breeding range in Japan (Tochigi 35.5°N 140.1°E, Chiba 35.8°N 140.1°E and Aichi 35.0°N 137.3°E; Fig 1), one site was located in the northern part (Iwate 39.2°N 141.2°E), and one in the southern part (Fukuoka 33.6°N 130.2°E). The studied extent of these sites ranged 66–143 km^2^. Investigations were conducted over a range of years: 5, 12, 11, 3, and 6 years, in Iwate, Tochigi, Chiba, Aichi, and Fukuoka, respectively. We used the nest-location data from the year with the maximum number of breeding nests recorded. Locations of these nests were recorded on topographical maps at 1:5,000 or 1:25,000 scales.

Vegetation was variable across the five sites. For example, the proportional area of all types of forests combined was high at one central region site, but low in another central region site (Chiba: 19%). Among forests, the proportion of deciduous broad-leaved forests was high in the northern region (S1 Table, S1 Fig, 62% of forests) and the northernmost site of the central region (Tochigi: 76%), while the proportion was low in the site of southern region (Fukuoka: 11%). The proportion of evergreen broad-leaved forests was high only in the site of the southern region (Fukuoka: 32%), likely due to the warmer climate.

The proportion of open-field habitats, such as paddy fields or grasslands, was high in one site of the central region (Tochigi: 59%) and in the southern region (Fukuoka: 57%), while low in another site of central region (Aichi: 9%). Among the open-field habitats, paddy fields dominated over grasslands in the northern region (82% of open-fields) and in two sites of the central region (Tochigi: 65%, Aichi: 85%). Paddy fields and grasslands shared similar proportions in one site of the central region (Chiba: paddy field 45%, grassland: 55%) and in the site of the southern region (Fukuoka: paddy field 49%, grassland: 51%).

Gray-faced buzzards are not an endangered species. In our study-sites, there were not any national park and other type of nature reserve that requires permissions to visit and conduct investigations. All five sites contain private lands. The following persons got agreements with the owners of the private lands to carry out the surveys in those study-sites: Atsuki Azuma (Iwate), Jun Nonaka (Tochigi), Yoshiaki and Hatsumi Sakai (Chiba), and Fumitaka Iseki (Fukuoka). Go Fujita got a permission to use data from the site of Aichi. The data were collected by Aichi Public Enterprise Bureau of Aichi Prefectural Government, which had an agreement with the owners to conduct surveys in the private lands. In observations carried out to locate breeding nests of the buzzards, all observers kept sufficient distances to the nests for minimizing disturbances to breeding buzzards. In addition, the observers did not visit around the nest sites during periods of early nest-building and egg-hatching in which there were relatively high possibilities of nest desertions.

### 2. Landscape Analyses

To measure landscape variables, such as areas or lengths of a landscape element, we used ArcGIS (Version 10.1) [26] and published datasets from the Vegetation Surveys for Natural Environmental Information [27, 28]. These vegetation maps were drawn based on maps at the 1:25,000 scale for four of the sites (Tochigi, Chiba, Aichi, and Fukuoka). For the site in Iwate, the only available vegetation map was at the 1:50,000 scale; therefore, we made a detailed vegetation map at this site based on both the published vegetation maps and a field survey in which we visited all the study sites and recorded boundaries of each vegetation type. By doing so, resolution of the boundaries became similar to that at the 1:25,000 scale vegetation map in other regions.

### 3. Calculating Nearest Neighbor Distances between Nests

As basic information of breeding distribution, we evaluated breeding densities of gray-faced buzzards at each site using the nearest neighbor distances (NNDs) between breeding nests. Nest locations were recorded on topographical maps in the field, and NNDs were measured using ArcGIS (Version 10.1).

### 4. Hypotheses Based on Regional Variation in Prey Items and Landscape Compositions

Here we focused on differences in prey items across regions that may alter the buzzard-landscape relationships. There are published and unpublished data on prey items fed to nestlings for four of the sites (Iwate: Azuma unpublished data, Tochigi: Momose et al. [16], Goshawk Protection Fund [23], Aichi: Matsuzawa et al. [22], and Fukuoka: Azuma, Iseki, and Itaya unpublished data). Using these datasets, we calculated Jaccard similarity coefficient of prey items for all pairs of sites (S2 Fig).

**Fig 2:**
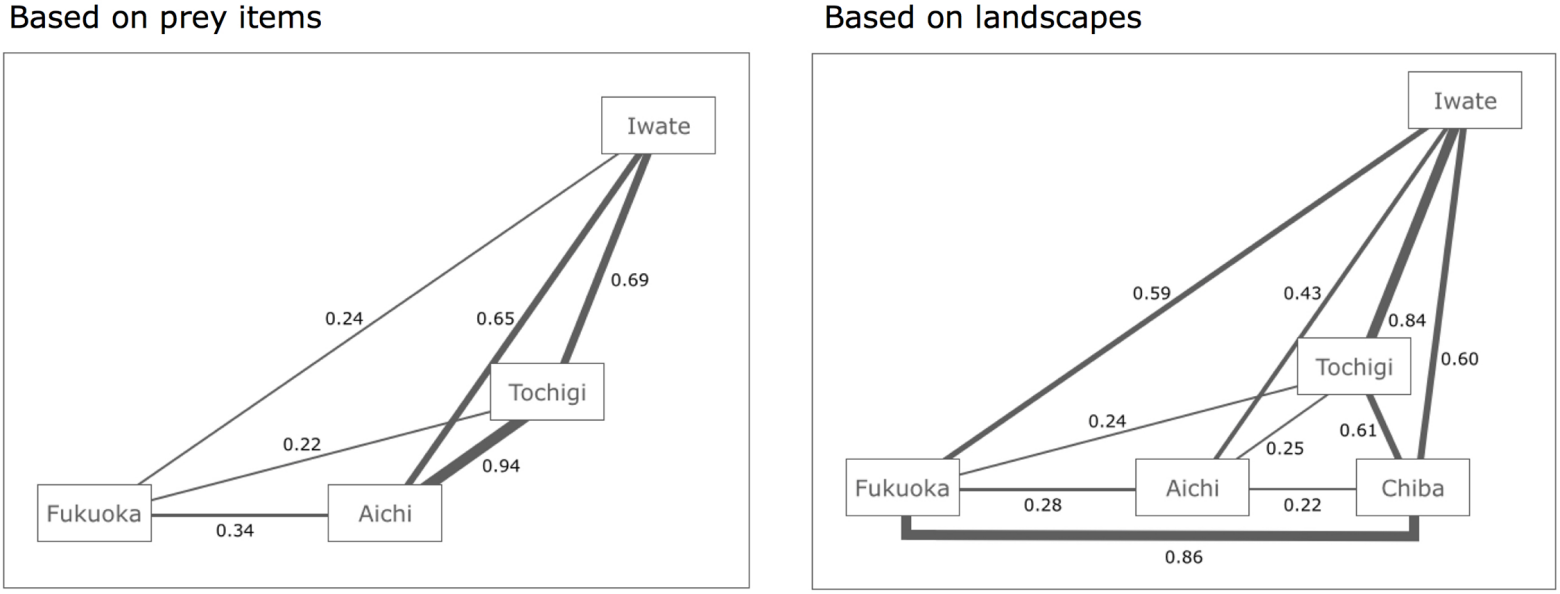
Jaccard similarity coefficient based on prey items and landscapes between study sites. Because there was no observation for prey items in Chiba, the indices on prey items were calculated between only four study sites.

The similarity coefficient of prey items was high between two of the sites of the central region (Tochigi–Aichi: 0.94), moderate between the site in the northern region and two sites of the central region (Iwate–Tochigi: 0.69, Iwate–Aichi: 0.65) (Fig 2 left panel), and low between the site of the southern region and the other sites (Fukuoka–Iwate: 0.24, Fukuoka–Tochigi: 0.22, Fukuoka–Aichi: 0.34). When looking at the difference in major prey items, the proportion of Lepidoptera was high only in sites of the central region. The proportion of frogs was high in sites of the central and northern regions, but extremely low in southern region. The proportion of small mammals increased with latitude, while the opposite trend was observed in lizards. Orthoptera and centipedes showed high proportions in the southern region.

We also calculated similarity indices based on landscape compositions (S1 Fig). The similarity coefficient of landscapes was high between Iwate–Tochigi (0.84) and Chiba–Fukuoka (0.86), and moderate between Iwate–Chiba (0.60), Iwate–Fukuoka (0.59), and Tochigi–Chiba (0.61) (Fig 2 right panel). These indices were low between Aichi and others (Aichi–Iwate: 0.43, Aichi–Tochigi: 0.25, Aichi–Chiba: 0.22, Aichi–Fukuoka: 0.28).

**Fig 3:**
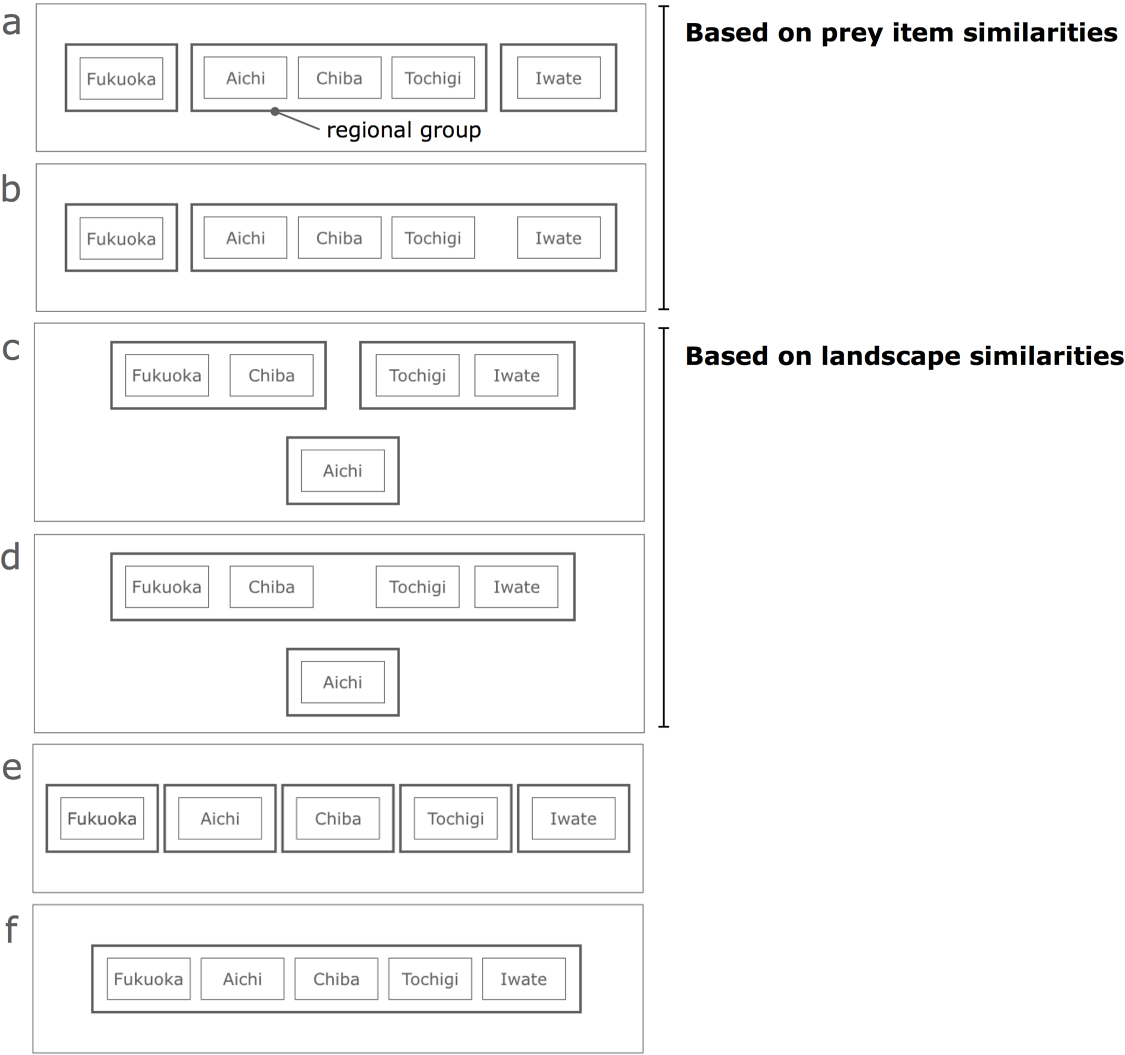
Schematic diagram representing six hypotheses to stratify five study-sites based on prey-item similarities (a, b), landscape-composition similarities (c, d), and others (e, f). **a:** 3 regional groups hypothesis based on prey item similarities, **b:** 2 regional group hypothesis based on prey item similarities, **c:** 3 regional group hypothesis based on landscape similarities, **d:** 2 regional group hypothesis based on landscape similarities, **e:** 5 regional group hypothesis, **f:** single regional group hypothesis.

We then established the following six hypotheses according to the above regional similarities in prey items and landscape compositions (Fig 3). Here we assumed that the buzzard-landscape relationships were similar when their prey items or landscape components show a high similarity.

1. **3 regional group hypothesis based on prey items** (Fig 3A)**:** the buzzard-landscape relationships are similar across the three central region sites (Tochigi, Chiba, and Aichi), while they are different across northern, central, and southern regions. Although there is no report on prey items in Chiba, we assumed that prey items in the area resemble those in Tochigi, because these two sites are the closest, geographically, of all sites, and within the same climate zone (Fig 1), in addition to similar habitat use in their breeding season [17, 18].
2. **2 regional group hypothesis based on prey items** (Fig 3B)**:** the buzzard-landscape relationships are similar between central sites and the northern site, and different between the southern site and all others.
3. **3 regional group hypothesis based on landscapes** (Fig 3C)**:** the buzzard-landscape relationships are similar between Iwate and Tochigi, and between Chiba and Fukuoka, and different between Aichi and all other sites.
4. **2 regional group hypothesis based on landscapes** (Fig 3D)**:** the buzzard-landscape relationships are similar across Iwate, Tochigi, Chiba, and Fukuoka, but different between Aichi and all other sites.
5. **5 regional group hypothesis** (Fig 3E)**:** the buzzard-landscape relationships are different across all five sites.
6. **Single regional group hypothesis** (Fig 3F)**:** the buzzard-landscape relationships are the same across all five sites.

### 5. Models

To examine these hypotheses, we used generalized linear models for the occurrence of breeding buzzards (1 km × 1 km grid cell), because this cell size is roughly equivalent to the home range size for buzzards, which covers an approximately 500 m radius from their nest [17]. Moreover, preliminary analyses showed that there were no statistical over-dispersions nor under-dispersions when we used this scale as a unit for the analysis.

For predictor variables, we used regional group ID, four types of landscape elements, and interaction terms between group ID and each of the landscape elements. It is important to note here that if a given interaction term is significant, it implies that buzzard-landscape relationship changes with regional difference in prey items or landscape compositions. The IDs for two-group hypothesis consisted of southern and other regions; IDs for three-group hypothesis consisted of southern, central, and northern sites; IDs for the five-group hypothesis used all five sites. Landscape elements included 1) areas of broad-leaved forests, 2) areas of grasslands, 3) areas of paddy fields, and 4) length of forest edge adjacent to grasslands and/or paddy fields. All of these landscape elements are known to be associated with major habitats for the buzzards' prey, according to previous studies on the foraging behavior of the buzzards [18, 29]. Orchards and cultivated areas were included under grasslands, because of the similarity of vegetation structures in Japan. Broad-leaved forests are likely to be a major habitat for Lepidoptera larvae, Orthoptera, and centipedes [30]. Grasslands are suspected to be preferred by lizards and snakes, while paddy fields are the major habitat for frogs [31]. We presumed that forest-edge is a suitable habitat for prey hunting, as it is a border of forests and open habitats that include grasslands and paddy fields [14, 18, 29]. We measured these landscape elements for each 1 km × 1 km grid cell using ArcGIS vegetation maps mentioned above.

To identify important predictor variables that explain the occurrence of buzzards, we conducted model selection using AICc (AIC with a correction for small sample sizes) [4]. When ΔAICc of models was ≤ 2, we regarded these as competing models and variables included in all these competing models were influential.

### 6. Reliability of Datasets

Because nests of buzzards are large and conspicuous, and observers were able to survey target habitats multiple times in a breeding season, possibilities of missing nests are likely to be minimal. The rationale is as follows. Firstly, all observers committed a large amount of time to search for active nest sites, i.e., observation effort in each site was 20–50 h/week during the breeding season (from April to late July). Second, in four of the five sites, nest-location surveys have been conducted over periods ranging over 5–13 years. In these cases, we used data from the year with the greatest number of recorded nests. In Aichi, surveys were conducted for only three years, with 160 h/week invested, and no new breeding cells were detected during the last two years (S3 Fig).

During the last three years of our observation, only limited numbers of cells were newly detected, as breeding cells, in four of the five sites (Tocihgi: 1, Chiba: 0, Aichi: 0, Fukuoka: 1). The Iwate site was an exception, i.e., we found three new breeding cells in 2011. Because we confirmed that observers invested a similar amount of survey effort between 2011 and the previous three years, these were designated as newly established nests in these areas.

## Results

Numbers of cells observed ranged from 66 in Tochigi to 143 in Iwate, and numbers of cells with one or more gray-faced buzzard nests were from 18 of Iwate in 2009 to 29 of Fukuoka in 2010 (see details in S2 Table). The total numbers of breeding nests found in our study sites in these years were from 20 in Iwate and Chiba (2012) to 32 in Fukuoka. Average NNDs between breeding nests varied across sites (S4 Fig). Fukuoka showed the smallest NND (0.69 ± 0.04 km) followed by Tochigi (0.73 ± 0.05 km). Iwate showed the largest NND (1.11 ± 0.13 km) followed by Aichi (1.01 ± 0.06 km). These results show that breeding densities were likely to be higher in Fukuoka and Tochigi, than in Iwate and Aichi.

**Fig 4:**
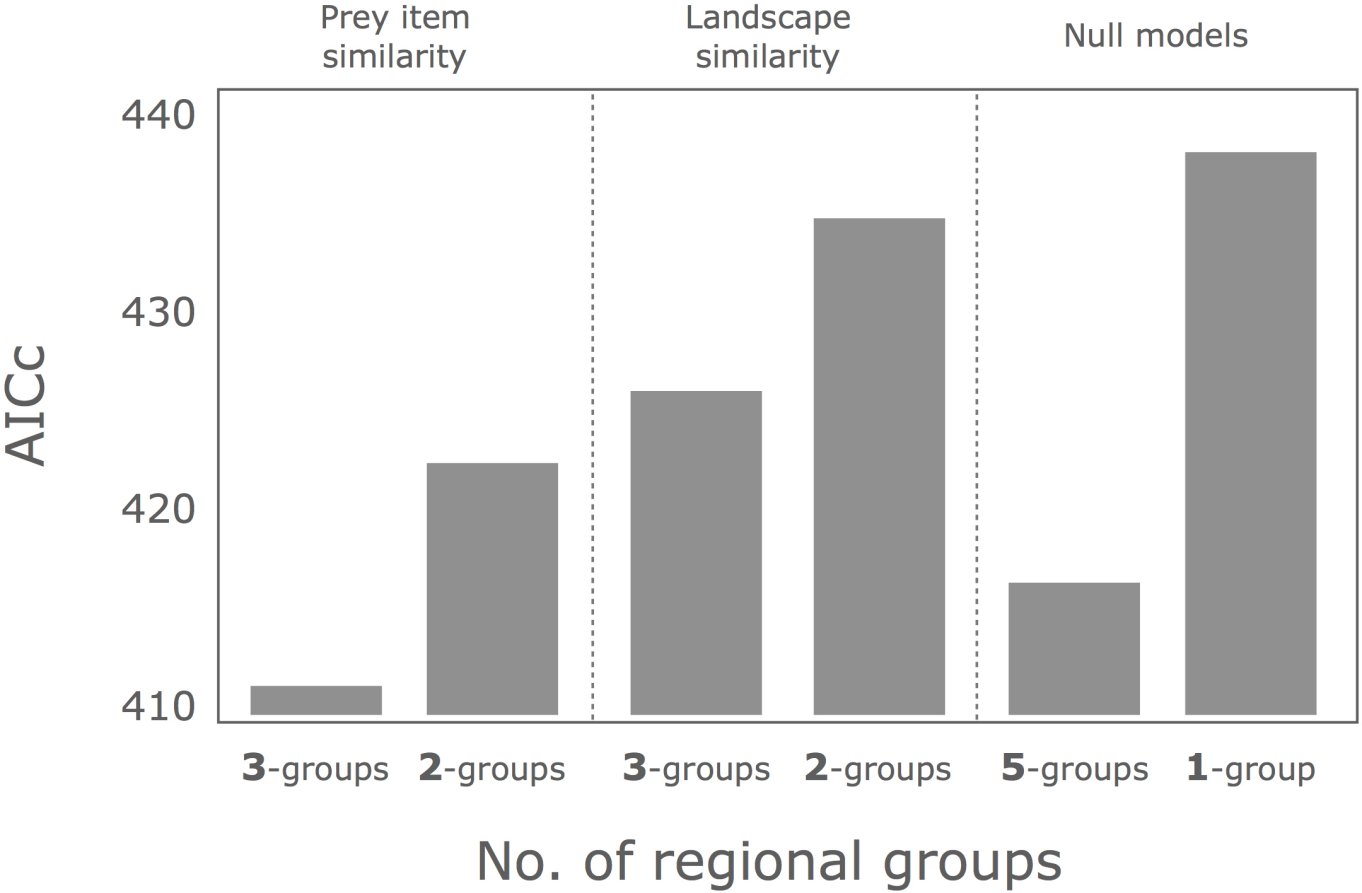
Values of AICc for the best models on hypothetical regional groups according to prey item or landscape similarities. Null model of 5-regional groups means all different study sites were different each other. Null model of 1-group means there was no difference among study sites.

Among possible hypotheses, the best model of 3 regional groups hypothesis based on similarity of prey items showed the smallest AICc (= 410.8, Fig 4, Tables 1–6). The best model of 5 regional groups hypothesis (AICc = 416.2) was the second, followed by 2 regional groups model based on prey-item similarity (AICc = 421.7). The best model of a single regional group hypothesis showed the largest AICc (= 437.8) among the six hypotheses. Within models for the 3 regional groups hypothesis, the ΔAICc value of the null model was 87.2, which showed little support for the null model relative to the best models.

**Table 1:** Selected models for 3 regional groups based on prey-item similarity (southern, central and northern) to explain relationships between buzzard occurrence and landscape elements.

Main effects					Interaction terms					
Forest edge	Broad-leaved forests	Grassland	Paddy field	Regional group ID	Edge x group ID	Forest x group ID	Grassland x group ID	Paddy x group ID	AICc	ΔAICc
4.36	1.67	4.08	-1.89	+	-	+	+	-	410.8	0.0
4.16	2.52	5.18	-	+	-	+	+	-	412.8	2.0
-	-	-	-	-	-	-	-	-	498.0	87.2

Figures (z values) and “+” show the predictor variables are included into selected models. Regional group IDs: Southern = a, central = b, northern = c.

**Table 2:** Selected models for 5 regional groups to explain relationships between buzzard occurrence and landscape elements.

Main effects					Interaction terms					
Forest edge	Broad-leaved forests	Grassland	Paddy field	Regional group ID	Edge x group ID	Forest x group ID	Grassland x group ID	Paddy x group ID	AICc	ΔAICc
3.39	2.54	2.39	-	+	-	+	+	-	416.2	0.0
3.50	1.77	1.91	-1.35	+	-	+	+	-	416.4	0.3
5.25	1.79	-	-2.28	+	-	-	-	-	417.1	1.0
4.20	1.29	-	-2.11	+	-	+	-	-	417.9	1.7
4.82	2.00	1.05	-1.80	+	-	-	-	-	418.1	2.0
-	-	-	-	-	-	-	-	-	498.0	81.8

Figures (z values) and “+” show the predictor variables are included into selected models. Regional–group IDs: Fukuoka = a, Iwate = b, Tochigi = c, Chiba = d, Aichi = e.

**Table 3:** Selected models for 2 regional groups based on prey-item similarity (southern and others) to explain relationships between buzzard occurrence and landscape elements.

Main effects					Interaction terms					
Forest edge	Broad-leaved forests	Grassland	Paddy field	Regional group ID	Edge x group ID	Forest x group ID	Grassland x group ID	Paddy x group ID	AICc	ΔAICc
4.73	1.70	1.90	-2.94	-4.21	-	-	-	-	421.7	0.0
5.52	-	1.46	-3.65	-4.22	-	-	-	-	422.4	0.8
6.52	-	-	-3.87	-5.44	-	-	-	-	422.5	0.8
4.68	1.32	1.95	-2.67	-4.24	-	-0.86	-	-	423.0	1.3
4.48	1.71	1.93	-2.16	-3.47	-	-	-	0.80	423.0	1.4
4.70	1.71	1.50	-2.82	-2.78	-	-	-0.80	-	423.1	1.4
6.17	1.16	-	-3.42	-5.54	-	-	-	-	423.2	1.5
1.33	1.66	1.91	-2.93	-3.73	0.42	-	-	-	423.5	1.9
-	-	-	-	-	-	-	-	-	498.0	76.3

Figures (*z* values) and “+” show the predictor variables are included into selected models. Regional-group IDs: Southern = a, central and northern = b.

**Table 4:** Selected models for 3 regional-groups based on landscape similarity (Aichi, Fukuoka-Chiba and Tochigi-Iwate) to explain relationships between buzzard occurrence and landscape elements.

Main effects					Interaction terms					
Forest edge	Broad-leaved forests	Grassland	Paddy field	Regional group ID	Edge x group ID	Forest x group ID	Grassland x group ID	Paddy x group ID	AICc	ΔAICc
3.00	-0.11	-1.20	-2.37	+	+	+	-	+	426.9	0.0
4.15	1.30	3.72	-	+	-	+	-	-	427.2	0.3
1.84	1.31	3.45	-	+	+	+	-	-	428.0	1.1
4.18	1.24	-1.09	-0.82	+	-	+	-	-	428.6	1.7
-	-	-	-	-	-	-	-	-	498.0	71.0

Figures (*z* values) and “+” show the predictor variables are included into selected models. Regional-group IDs: Aichi = a, Fukuoka and Chiba = b, Tochigi and Iwate = c.

**Table 5:** Selected models for 2 regional-groups based on landscape similarity (Aichi and others) to explain relationships between buzzard occurrence and landscape elements.

Main effects					Interaction terms					
Forest edge	Broad-leaved forests	Grassland	Paddy field	Regional group ID	Edge x group ID	Forest x group ID	Grassland x group ID	Paddy x group ID	AICc	ΔAICc
3.02	1.70	-1.20	-2.48	1.76	-2.52	-	1.37	2.32	436.2	0.0
2.93	1.54	3.72	-2.28	1.49	-2.25	-	-	2.06	436.5	0.4
3.19	-	3.45	-2.63	1.88	-2.38	-	-	2.32	436.8	0.6
3.12	-	-1.09	-2.64	1.76	-2.54	-	1.23	2.43	436.9	0.8
3.52	1.72	4.05	-2.78	-	-	-	-	-	437.8	1.6
3.05	0.18	-1.11	-2.52	1.75	-2.56	0.54	1.28	2.37	438.0	1.8
2.98	-0.12	3.78	-2.35	1.63	-2.34	0.81	-	2.15	438.0	1.8
-	-	-	-	-	-	-	-	-	498.0	61.8

Figures (*z* values) and “+” show the predictor variables are included into selected models. Regional-group IDs: Aichi = a, others = b.

**Table 6:** Selected models for single regional-group.

Forest edge	Broad-leaved forests	Grassland	Paddy field	AICc	ΔAICc
3.52	1.72	4.05	-2.78	437.8	0.00
4.37	-	3.74	-3.45	438.6	0.86
-	-	-	-	498.0	60.20

Figures show *z* values.

**Fig 5:**
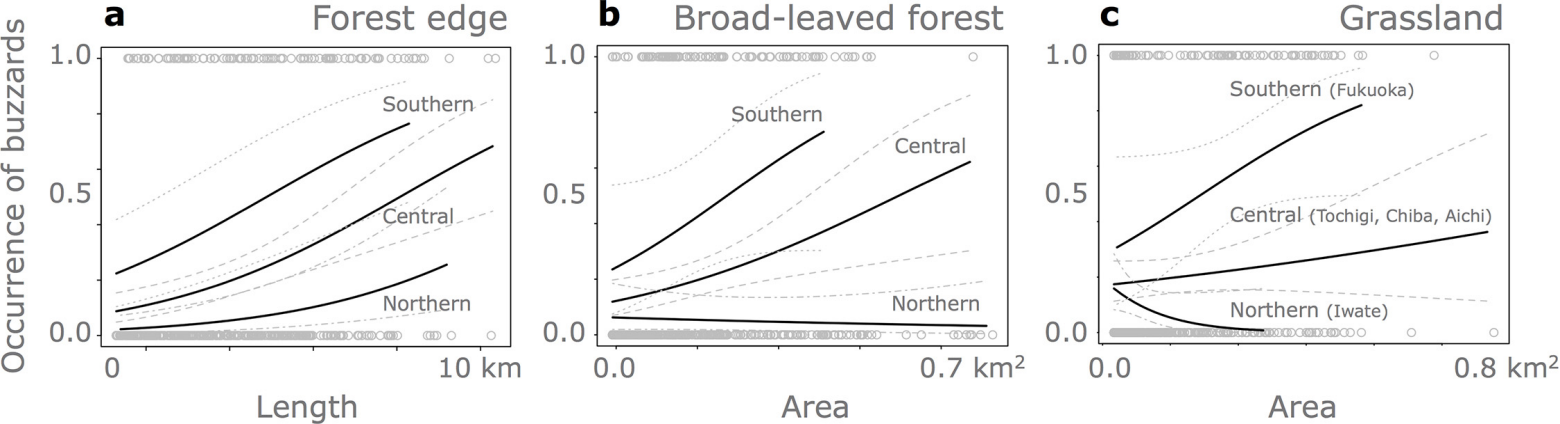
Relationships between buzzard occurrence and landscape elements (forest-edge, broad-leaved forests, and grasslands) in 1 km x 1 km cells inferred by the selected models for the three geographical-regions hypothesis. Solid lines show inferred relationships. Dotted, dashed, and chain lines represent 95% confidence intervals for the relationships in southern, central, and northern regions, respectively. Open circles are observed values.

In the competing models of 3 regional groups models, length of forest-edge, areas of broad-leaved forest and grassland, as well as the regional group ID were included as the main effects, in addition to the interaction terms of broad-leaved forests x group IDs and grasslands x group IDs. In these models, the coefficient of forest edge was consistently positive (Fig 5A). As the interaction term with group IDs were selected for areas of both broad-leaved forests and grasslands, buzzard-landscape relationships (i.e. occurrence of buzzards vs. landscape elements in 1 km x 1 km cells) were different among 3 regional groups, and a positive relationship was found in central and southern groups whereas no or weak relationship in northern group (Fig 5B and 5C).

These results suggested that grouping of study-sites based on similarities of prey items is most effective for explaining how buzzard-landscape relationships change across regions. That is, these relationships were similar among three study-sites in central regional group (Tochigi, Chiba and Aichi), while the relationships in northern (Iwate) and southern (Fukuoka) groups were largely different from those in central regional group. Specifically, the positive association between buzzards and broad-leaved forests, and that between buzzards and grasslands became clearer as moving southward.

## Discussion

We showed regional differences in habitat models for an apex predator, gray-faced buzzards, which breeds in paddy field dominated landscapes, at a landscape scale. Results showed that 3 regional groups, defined by prey-item similarities of the buzzards, best explained the occurrence of buzzards. These 3 regional groups consisted of northern (Iwate), central (Tochigi, Chiba, and Aichi), and southern (Fukuoka) regions. In addition, we found a clearer, positive relationship between buzzard occurrence and landscapes (grasslands and broad-leaved forests) along a southward gradient.

A possible mechanism causing these regional differences is the difference in prey availability between grasslands and broad-leaved forests. Major prey for gray-faced buzzards are arthropods such as grasshoppers and Lepidoptera larvae, i.e. caterpillars, and small vertebrates such as frogs and skinks (see S2 Fig) [16, 19–23]. In the northern region, these arthropods and vertebrates are not very active during the breeding season of the buzzards (from late April to early July) because they are poikilotherms. When cold Pacific winds or ‘Yamase’ blow, temperature decreased drastically [32]. However, they are likely to be abundant in early spring in the southern region [30, 31] because of its warmer climate (e.g., 8.6°C in Iwate, 12.9°C in Chiga, and 14.0°C in Fukuoka) [32]. In addition, some grasshoppers and katydids overwinter in their adult stage, occasionally without hibernation [30], and their species richness is greater in the southern region [30]. Such high diversity of prey may keep available prey densities at higher levels throughout a season, due to the complementation of emergence peaks across species.

In Japan, major habitats for prey arthropods (grasshoppers and katydids) and reptiles (Japanese five-lined skinks and green grass lizards) are known to be grasslands and broad-leaved forests [30, 31]. Thus, buzzard prey densities are expected to increase in grasslands and broad-leaved forests along a southward gradient, and these could strengthen the buzzard-landscape relationship in southern regions.

Another possible factor that may strengthen the relationship between buzzards and grasslands or broad-leaved forests, in the southern region, is the decreased importance of paddy fields as foraging habitats there. Although paddy fields are an important habitat for frogs, a major prey of buzzards in the central region [31, 33], densities of frogs may be much lower in the southern region than in the central region, due to the delayed timing of flooding in paddies, and rice-planting (late June, Iseki pers. obs. Misako Kuroe unpublished data). This delay is caused by the double cropping system common in the southern region [34], i.e., cultivation of soybean and wheat, before rice planting. As a result, the importance of grasslands and broad-leaved forests, relative to paddy fields, may have an increased importance for buzzards in southern region.

Because we have no data on prey availability, we presume that prey compositions (S2 Fig) more or less reflected prey availability in each site. Further studies on prey preferences of the buzzards with considerations of prey availability are required.

Other than prey availability, presence of other raptor species could have affected the buzzard-landscape relationship through competition with gray-faced buzzards. In general, sympatric raptors often establish inter-specific territories [35, 36], and dominant raptor species monopolize suitable habitats, leading to exclusion of subordinate raptor species [35]. In addition, intra-guild predation among apex predators could alter habitat use of those species [37]. These could weaken the relationship between the occurrences of a subordinate raptor species and landscape characteristics.

In our southern region site, black kites *Milvus migrans* were recorded as the only breeding raptors other than gray-faced buzzards while in the sites located in the central and northern regions, goshawks *Accipiter gentilis*, eastern buzzards *Buteo japonicas* and common kestrels *Falco tinnunculus* were recorded as possible breeding raptors in addition to black kites. Dominance hierarchies among the predators are not well known, but intraguild predation by goshawks on gray-faced buzzards were observed in Tochigi, one of the central sites (Nonaka unpublished data). In addition, density of eastern buzzards was much higher in the northern region (0.7/km^2^) than in the central region (0.1/km^2^), and they sometimes took over breeding territories from gray-faced buzzards (Azuma and Nonaka unpublished data). These suggest that the presence of eastern buzzards may be responsible for the weak relationship between gray-faced buzzards and landscapes, in the northern region.

It is probable that there are regional differences in average home range size due to, e.g., differences in landscape compositions. Under such conditions, underestimates of suitable habitats because 1 km^2^ cells with suitable habitats are likely to contain multiple nests where the average home range sizes are small, less than 1 km^2^. Among our study sites, we might have possibilities of underestimates of suitable habitats in Fukuoka and Tochigi with relatively small NNDs between nests though differences in the NNDs were not large (S4 Fig).

Earlier studies have revealed regional differences in species-landscape relationships, although the mechanisms were unclear (e.g., farmland birds in south England and Wales [38], koala in southeast Australia [39]). These studies proposed that habitat management strategies should not be identical across a nationwide scale. However, several conservation practices implicitly assume that a given management practice, for a target species, has a similar effect across regions [1]. This is probably because the establishment of habitat models in each region is not realistic, especially for endangered species with a large distribution range and a low density [40, 41]. An efficient way to resolve this problem would be to identify the ecological mechanisms underpinning regional difference in species-landscape relationships. Here we showed that regional differences in prey items used by gray-faced buzzards appear to be related to the regional differences in the buzzard-landscape relationship. We suggest that information on prey organisms may be the key to predict model transferability for top predators.

Although collecting data on prey species can be laborious, such information is occasionally available for apex predators, such as birds of prey (e.g., sparrowhawks [42], common buzzards [43]) and mammalian predators (e.g. introduced canine species in Australia: Davis et al. [44]), probably because they attract high levels of human interest. It is possible, therefore, to provide suitable implications of conservation practices, with knowledge of the ecological mechanisms generating regional differences in habitat models.

In addition, to improve the current situation in which these data are only occasionally available, monitoring of prey items is recommended to include into action plans for conservation strategy by government organizations or civilian scientists.

### Implications for Management Practice of Gray-Faced Buzzards

Previous studies on habitat models of gray-faced buzzards stressed the importance of forest-edge [16, 29], but this opinion is based on studies carried out in several sites located in the central regions of the breeding range (one of the studies included our site in Tochigi). Here we have confirmed that forest-edges had positive correlations throughout the five sites, located from the northern edge to the southern area of the breeding range in Japan. However, we also showed that habitat models of buzzards vary among regions, and described how the importance of grasslands and broad-leaved forests are increased in southern regions. This suggests that a landscape consisting of a high proportion of grasslands and/or broad-leaved forests is likely to be suitable for buzzard breeding, as well as a landscape with a large amount of forest-edges. Especially in areas near large cities, broad-leaved forests are being fragmented because of urbanization [45], while grasslands have been drastically decreased due to farmland abandonments, particularly in rural areas [45]. As gray-faced buzzards are decreasing in the last several decades in Japan [14], it is necessary to identify spatially explicit areas that are suitable for breeding, with consideration of regional difference in habitat suitability as revealed in this study.

## Supporting Information

S1 Fig Landscape compositions in five study sites for inferring habitat models of gray-faced buzzards in Japan. (TIFF)

S2 Fig Proportions of prey items fed on chicks of gray-faced buzzards in four studied regions in Japan. All those records were carried out using video cameras set close by the nests. Records by the cameras were conducted throughout the nestling periods.
(TIFF)

S3 Fig Cumulative curves of cell numbers where breeding sites of buzzards were detected in five study sites for last three observation-years. (TIFF)

S4 Fig Average nearest neighbor distances between gray-faced buzzard nests in five study-sites of Japan. Error bar shows standard error.
(TIFF)

S1 Table Proportions in areas of all types of forests and open-fields in five study sites. Proportions of deciduous and evergreen broad-leaved and planted coniferous forests within all types of forests, and those of paddy fields and grasslands within open-fields are also shown.
(XLSX)

S2 Table Maximum numbers of used-cells, numbers of breeding nests in a year when the used-cells were the maximum, number of studied cells, and year when the used-cells were maximum in each study sites. (XLSX)
