# Supplementary figures and images for: Context Dependent Effect of Landscape on the Occurrence of an Apex Predator across Different Climate Regions
Source: PLoS One. 2016 Apr 28;11(4):e0153722. doi: 10.1371/journal.pone.0153722 (PMC4849657; doi:10.1371/journal.pone.0153722)

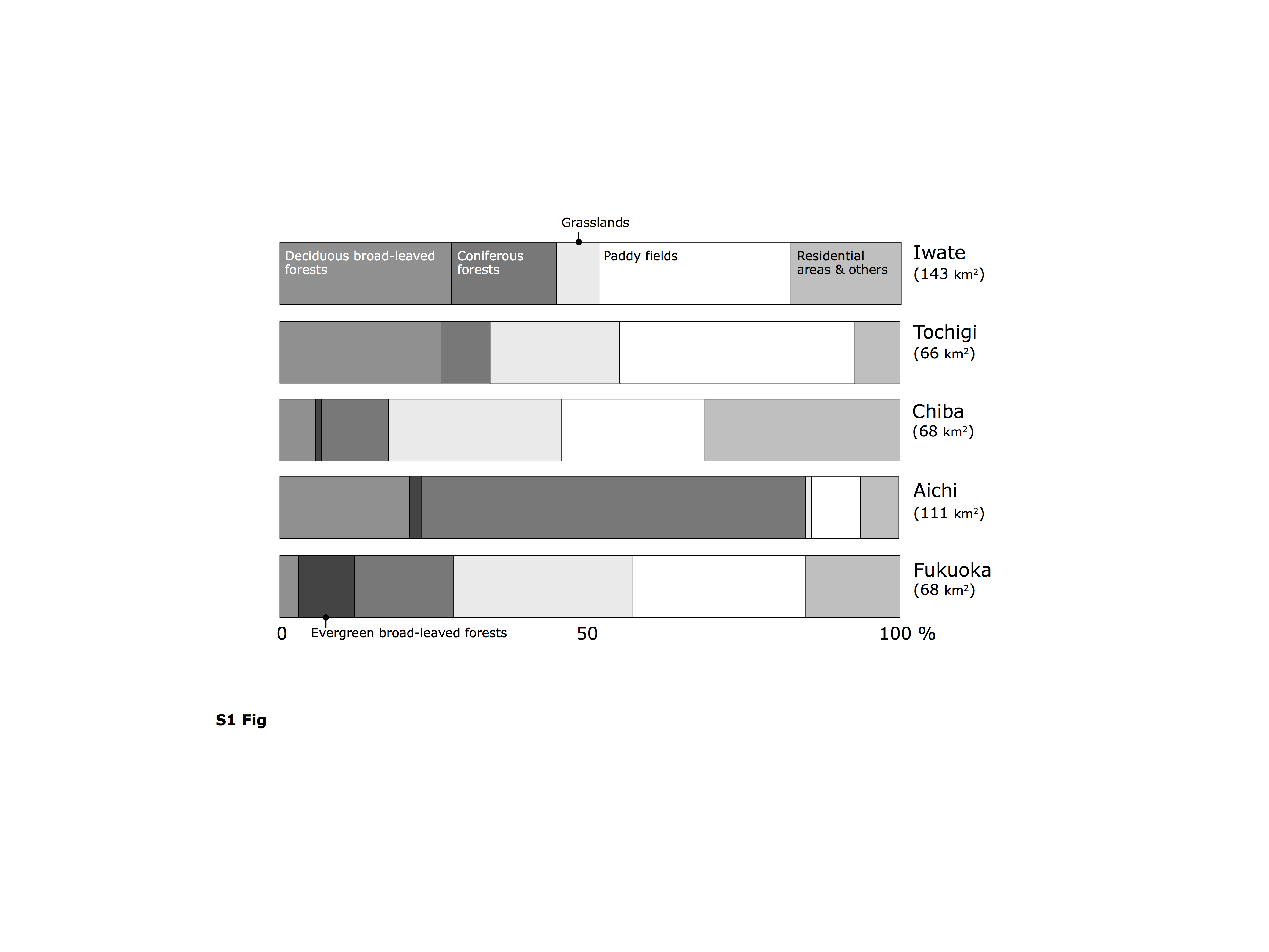

Supplement: S1 Fig — (TIFF) [file pone.0153722.s001.tiff]

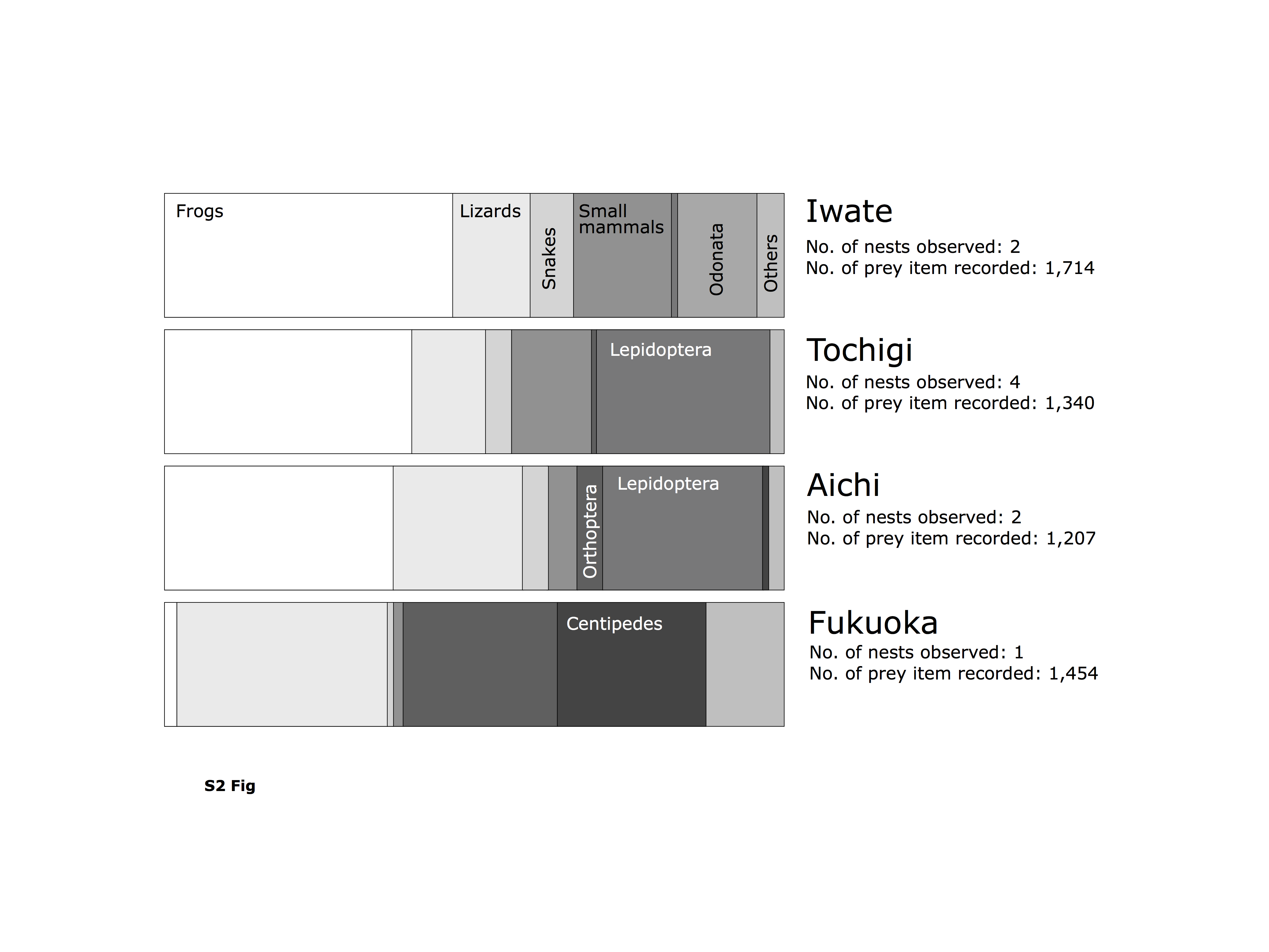

Supplement: S2 Fig — All those records were carried out using video cameras set close by the nests. Records by the cameras were conducted throughout the nestling periods. (TIFF) [file pone.0153722.s002.tiff]

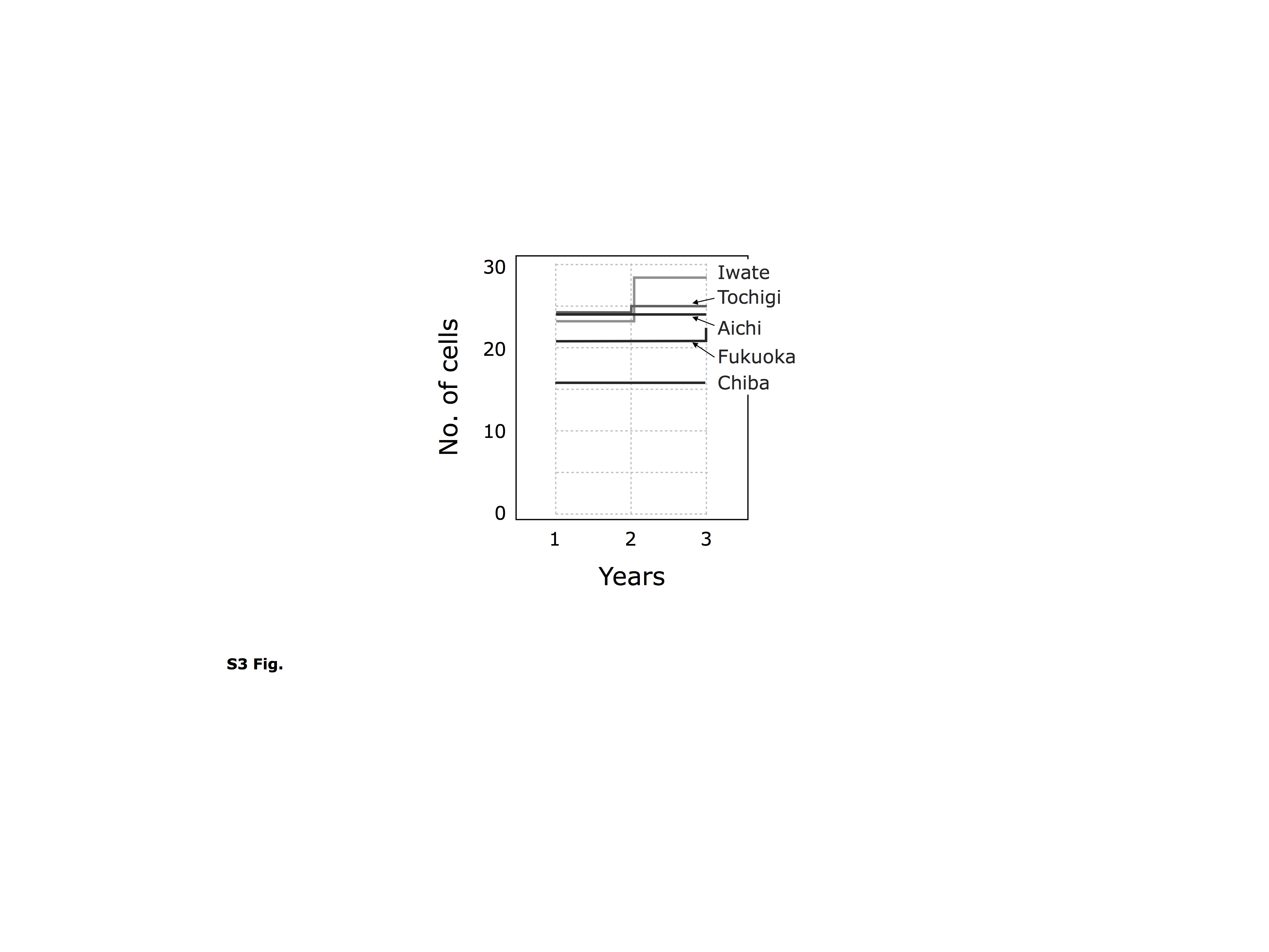

Supplement: S3 Fig — (TIFF) [file pone.0153722.s003.tiff]

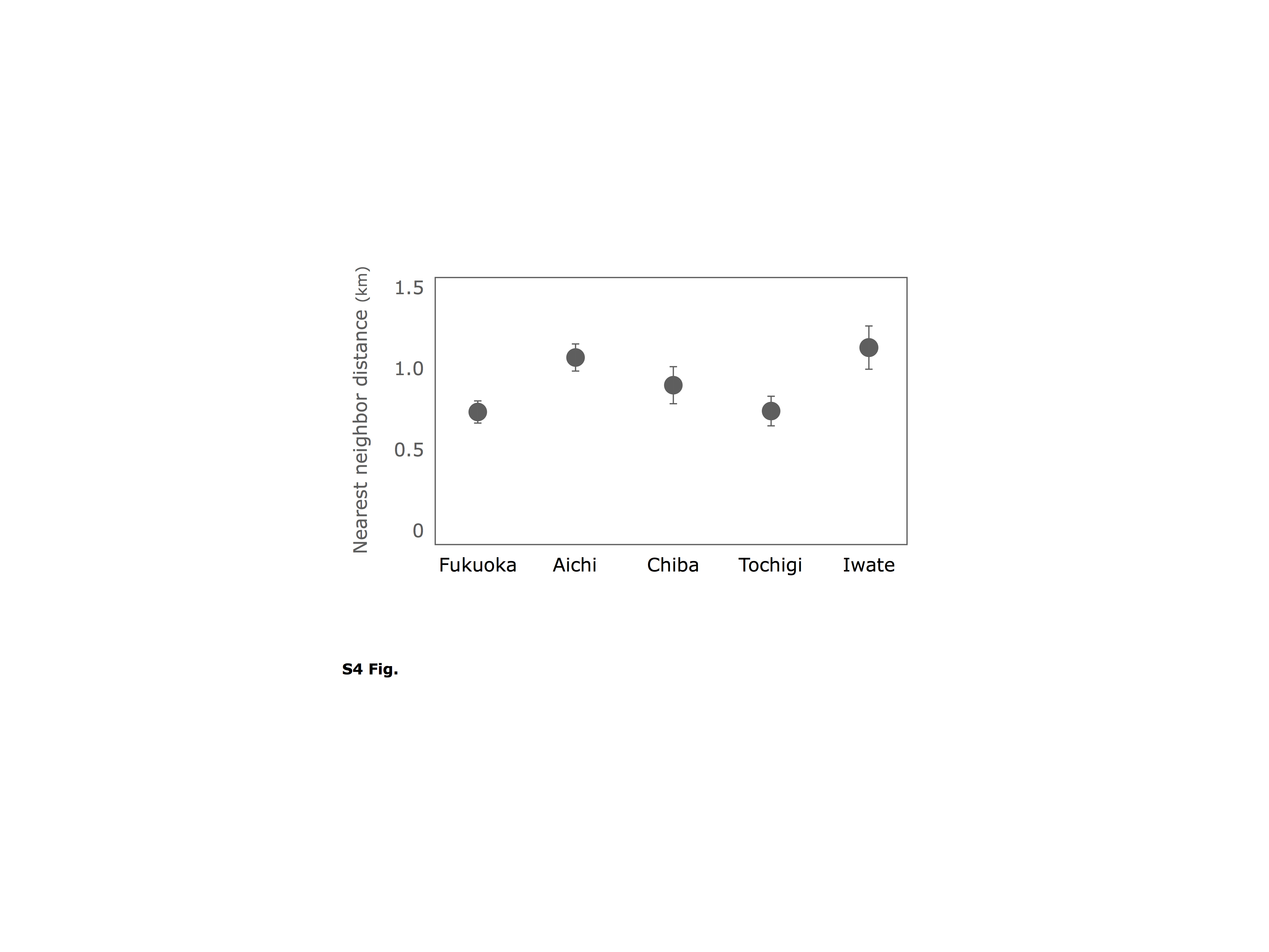

Supplement: S4 Fig — Error bar shows standard error. (TIFF) [file pone.0153722.s004.tiff]
